# The YoaW signal peptide directs efficient secretion of different heterologous proteins fused to a StrepII-SUMO tag in *Bacillus subtilis*

**DOI:** 10.1186/s12934-019-1078-0

**Published:** 2019-02-07

**Authors:** Janine Heinrich, Chris Drewniok, Eva Neugebauer, Harald Kellner, Thomas Wiegert

**Affiliations:** 1 Department of Microbiology, Faculty of Natural and Environmental Sciences, University of Applied Sciences Zittau/Görlitz, Theodor-Körner-Allee 16, 02763 Zittau, Germany; 2 EUROIMMUN AG, Im Kreppel 1, 02747 Herrnhut/Rennersdorf, Germany; 3 Department of Bio- and Environmental Sciences, International Institute Zittau, Technical University of Dresden, Markt 23, 02763 Zittau, Germany

**Keywords:** *B. subtilis*, SUMO, Secretion, Heterologous expression

## Abstract

**Background:**

Heterologous gene expression is well established for various prokaryotic model systems. However, low yield, incorrect folding and instability still impede the production of soluble, bioactive proteins. To improve protein production with the Gram-positive host *Bacillus subtilis*, a secretory expression system was designed that enhances translocation, folding and stability of heterologous proteins, and simplifies purification. Based on the theta-replication plasmid pHT01, a *B. subtilis* secretory expression vector was constructed that encodes a fusion protein consisting of a signal peptide and a StrepII-tag linked to a SUMO-tag serving as a folding catalyst. The gene of a protein of interest can be translationally fused to the SUMO cassette and an additional 6xHis-tag encoding region. In order to maximize secretory expression of the construct by fitting the signal peptide to the StrepII-SUMO part of the fusion protein, a *B. subtilis* signal-peptide library was screened with the *Escherichia coli* alkaline phosphatase PhoA as a reporter.

**Results:**

The YoaW signal peptide-encoding region (SP*yoaW*) was identified with highest secretory expression capacity in context with the StrepII-SUMO-tag fusion in a *B. subtilis* eightfold extracellular protease deletion strain. PhoA activity and fusion protein production was elevated by a factor of approximately five when compared to an α-amylase (AmyQ) signal peptide construct. Replacement of PhoA with a single-chain variable fragment antibody specific for GFP or the *B. amyloliquefaciens* RNase barnase, respectively, resulted in a similar enhancement of secretory expression, demonstrating universality of the YoaW signal peptide-StrepII-SUMO encoding cassette for secretory expression in *B. subtilis*. Optimisation of codon usage and culture conditions further increased GFP-specific scFv fusion-protein production, and a simple affinity purification strategy from culture supernatant with removal of the StrepII-SUMO-tag by SenP-processing yielded 4 mg of pure, soluble and active GFP-specific scFv from 1 l of culture under standard laboratory conditions.

**Conclusions:**

The new expression system employing a YoaW signal peptide-StrepII-SUMO fusion will simplify secretory protein production and purification with *B. subtilis.* It can obviate the need for time consuming individual signal-peptide fitting to maximize yield for many different heterologous proteins of interest.

**Electronic supplementary material:**

The online version of this article (10.1186/s12934-019-1078-0) contains supplementary material, which is available to authorized users.

## Background

For decades, Gram-positive *Bacillus* species have been widely used as prokaryotic hosts for the production of secretory proteins in industrial biotechnology. The absence of an outer membrane barrier enables proteins to be exported from the cytoplasm directly in the culture medium. Moreover, toxic by-products like pyrogenic lipopolysaccharides are not formed, qualifying *Bacillus* strains as safe for food and feed applications. Due to the high secretion capacity of *Bacillus* strains, hydrolytic enzymes like proteases, amylases and lipases, especially those originating from *Bacillus* species or close relatives, are generated at a scale of several grams per liter in culture supernatant and can be isolated without time and cost intensive cell rupture in downstream processing [1].

Unfortunately, these high yields of grams per liter are not achievable for recombinant production of most other proteins. For example, antibodies or antibody fragments originating from higher eukaryotes can only be recovered at low levels in the range of milligrams per liter of culture (e.g. [2]). The simple and low-cost fermentation strategies at a large scale that are well established for *Bacillus* strains could satisfy the strong demand for inexpensive proteins of pharmaceutical relevance. Therefore, many efforts aimed to improve *Bacillus* strains as secretory production hosts. It turned out that among a multiply of reasons, high level secretory production seems to be limited by three main characteristics that render foreign proteins incompatible to the *Bacillus* production host, affecting translocation, folding and stability (reviewed in [3–5]).

First, translocation from the bacterial cell has to be accomplished by fusing the foreign protein of interest (POI) to a secretion signal, most commonly to a signal peptide of the general SEC secretion pathway. A variety of expression plasmids encoding different signal peptides have been constructed for secretory expression of POIs with *Bacillus,* e.g. with signal peptides from *Staphylococcus* Protein A [6], from *B. amyloliquefaciens* alkaline protease, neutral protease, barnase, levansucrase [7] and amylase [8], and from *B. subtilis* lipase A [9]. However, it became apparent that the signal peptide must fit to the POI to maximize expression and to allow optimal interaction with the secretion machinery [5]. So far, theoretical predictions for fitting signal peptides to POIs are not available, and in practice, a time consuming and work-intensive screening for a best-fitted signal peptide has to be performed [5].

Second, successful translocation is completed by proper folding of the secreted POI, which is important to gain its native structure as well as prevent blocking of the translocation machinery by forming illegitimate interactions with the cell wall or by forming insoluble aggregates through intermolecular interactions [3]. With *E. coli,* secretory expression vectors allowing translational fusions to the maltose binding protein (MBP), which has been proposed to exhibit chaperone activity [10], are used to that purpose.

Third, extensive degradation of the foreign protein by the host’s extracellular proteases has to be avoided. *B. subtilis* produces a whole bunch of different proteases active in the extracytoplasm, and the use of strains with multiple deletions in extracellular protease genes is absolutely necessary [11–13].

When we tried to express different genes with the commercial available vector pHT43 for secretory protein production (Mobitec GmbH, Göttingen, Germany) in the recommended *B. subtilis* eightfold extracellular protease deficient strain WB800N [14], sometimes only poor results in the range of micrograms per liter of culture medium were achieved. Therefore, we decided to realize a system that might improve and simplify secretory protein production in *B. subtilis* by addressing the three main obstacles described above, i.e. secretion, folding and stability.

**Fig. 1:**
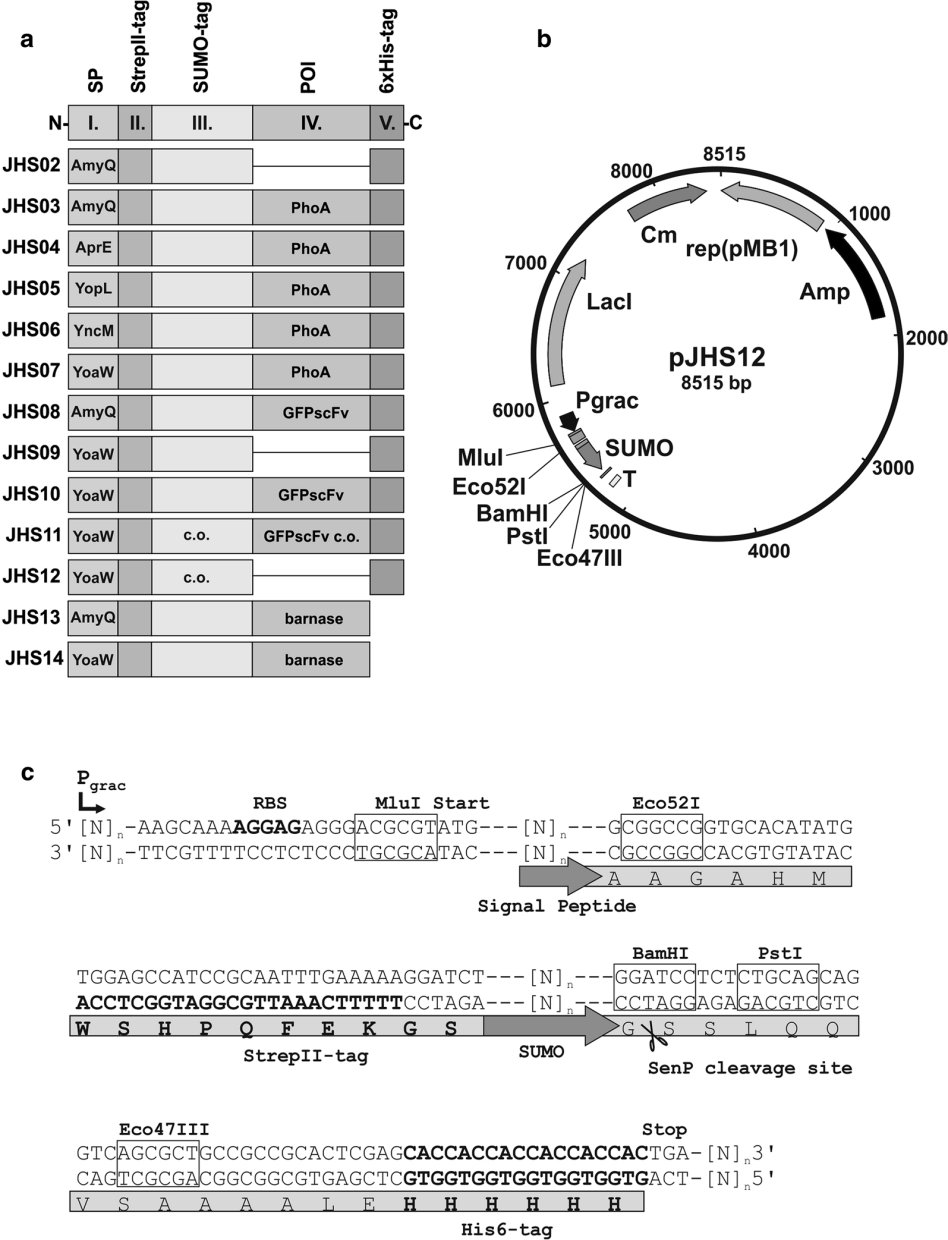
Schematic representation of the pJHSP expression vector system. **a** Schematic representation of fusion proteins JHS02–JHS14 encoded on plasmids pJHS02–pJHS14 that include an N-terminal signal peptide (SP) for SEC-dependent secretion (I.) followed by a StrepII-tag (II.) and the SUMO protein (III.). The protein of interest (POI; IV.) is fused to the C-terminus of SUMO, and to the N-terminus of a 6xHis-tag (V.). c.o.: encoding DNA-sequence codon optimized for *B. subtilis*. **b** Map of plasmid pJHS12. Cm: chloramphenicol resistance gene, Amp: ampicillin resistance gene, Rep(pMB1): origin of replication, LacI: Lac repressor gene. The SUMO fusion cassette is controlled by the Pgrac promotor, and a transcriptional terminator (T) sequence is placed at the 3′ end. **c** Detail of pJHS12 showing relevant sequences of the reporter fusion cassette. A signal peptide-encoding sequence can be inserted in the unique MluI and Eco52I restriction sites for an in frame fusion to the StrepII-SUMO part. A gene of interest can be fused via the unique BamHI, PstI and Eco47III restriction sites with the option to add a C-terminal polyhistidine tag. The SenP-processing site is indicated by scissors. RBS: ribosome binding site

As a vector, we used a structurally stable, theta replicating plasmid for *B. subtilis* that allows overexpression of inserted genes from an IPTG regulatable promotor [8]. A translational fusion was cloned to that vector, encoding, from N- to C-terminus, a signal peptide of the SEC translocation pathway (I.), a StrepII-tag for affinity purification (II.), a SUMO-tag as folding catalyst (III.) and a 6xHis-tag for affinity purification (V.; compare Fig. 1a). The POI encoding region (IV.) is inserted between SUMO and polyhistidine-tag. When expressed in *B. subtilis*, the translated fusion protein will be secreted to the medium and the signal peptide (I.) will be cleaved off by signal peptidase, resulting in an extracellular fusion protein consisting of StrepII-tag, SUMO-tag, POI, and (optional) 6xHis-tag. SUMO stands for the ‘small ubiquitin related modifier’ protein, the normal function of which is post-translational modification in eukaryotes. For heterologous gene expression, it serves as an established fusion-tag of about 80 amino acid residues in length that enhances production, folding and stability of proteins connected, similar to MBP [15]. One major advantage in using SUMO is the possibility to remove the tag via SenP-protease, which recognizes the three dimensional structure of SUMO instead of a sequence motif and, therefore, enables formation of an authentic N-terminus of the POI after processing [16]. N-terminal polyhistidine-tag and C-terminal StrepII-tag allow a fast and easy affinity-purification strategy from culture supernatants (Additional file 1: Fig. S1A) using immobilized metal ion- and streptactin-affinity chromatography, resulting in a highly pure protein product without degradation products (Additional file 1: Fig. S1B). Using an *E. coli* alkaline phosphatase (PhoA) reporter screen, we then identified a SEC signal peptide from a library of 173 *B. subtilis* signal peptides that maximizes expression and secretion in connection with the StrepII-SUMO part, exemplified by a single chain variable fragment antibody against the green fluorescent protein (GFP-specific scFv). Furthermore, we show that strain WB800N is not advisable as a production host together with most of the *E. coli/B. subtilis* shuttle vectors, as whole genome sequencing of the strain revealed a cryptic *E. coli* plasmid inserted in its chromosome, which implies the risk of unfavorable recombination.

## Results

### Genome-analysis of the eightfold protease deletion strain *B. subtilis* WB800N

One of the most important prerequisites for secretory expression of heterologous proteins with *B. subtilis* is to reduce extracellular protease activity. The eightfold extracellular protease deletion strain WB800 [2] is widely used to reduce degradation of secreted proteins. Strain WB800 contains a chloramphenicol resistance marker (*cat*) at unknown position, and its descendant strain WB800N includes a neomycin resistance cassette (*neo*) within the *cat* resistance marker [14]. To localize the resistance markers and to verify protease gene-deletions, we performed Ion Torrent whole genome DNA-sequencing that yielded 2.7 million reads. After filtering, 2.2 million reads were used for re-mapping and de-novo assembly. The de-novo assembly resulted in 13 contigs (Acc. No. SRP155925), one of which reveals the *cat::neo* cassette. Surprisingly, the contig encompasses an additional β-lactamase-resistance marker (*bla*), a major part of the tetracycline resistance gene *tetA* and the *E. coli* ColE1 origin of replication. The contig is flanked by genomic *B. subtilis* sequences of the *rsbRB* and the *metE* gene. As meanwhile a markerless clean deletion strain of seven extracellular proteases was available (*B. subtilis* KO7; D. Zeigler unpublished), we refrained in using WB800N in further studies. We combined the *wprA::hyg* deletion of WB800 with KO7 to obtain *B. subtilis* KO7A, which is deleted for the same eight extracellular proteases as WB800.

### Construction of a secretory PhoA reporter tagged with SUMO and identification of signal peptides for high secretion expression in *B. subtilis*

**Fig. 2:**
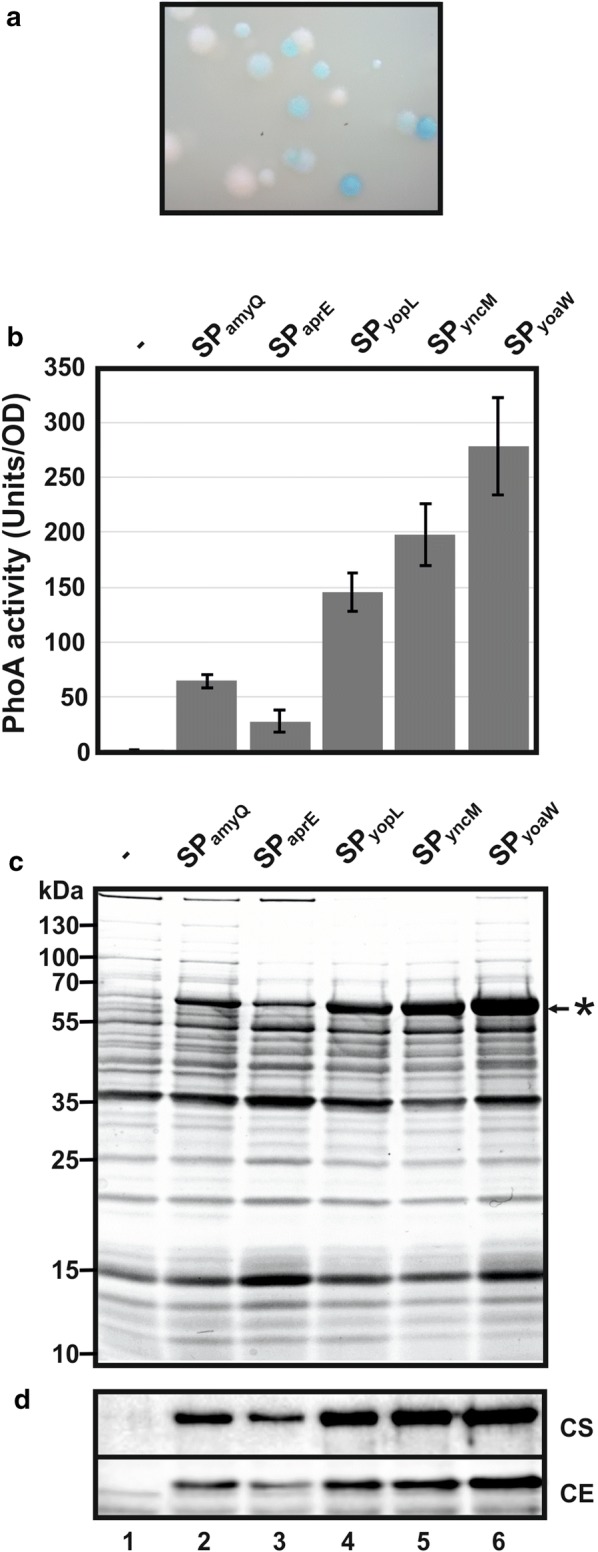
Identification of signal peptide sequences for enhanced secretory expression of SUMO-fused heterologous proteins in *Bacillus subtilis.* **a** Visualisation of alkaline phosphatase activity of representative *B. subtilis* KO7A colonies on solid medium containing the chromogenic substrate BCIP and IPTG as an inductor of PhoA reporter-gene expression. The colonies derived from a transformation of *B. subtilis* KO7A with plasmid pJHS03 with the SP*amyQ* encoding sequence replaced by a DNA-library of 173 different *B. subtilis* signal peptide encoding sequences. **b** Extracellular PhoA activities of three *B. subtilis* KO7A clones isolated from the signal peptide screen. KO7A/pJHS02 served as a negative control (−), KO7A/pJHS03 (SP*amyQ*) and KO7A/pJHS04 (SP*aprE*) served as positive controls. The signal peptides were identified as SP*yopL* (pJHS05), SP*yncM* (pJHS06) and SP*yoaW* (pJHS07) by DNA sequencing of re-isolated plasmids. IPTG was added to each 50 ml bacterial culture shaking at 37 °C at a final concentration of 1 mM at an OD_600_ of 0.8. Samples were withdrawn after 4 h and PhoA activities were measured as described in “Methods” section. **c** In parallel to PhoA activity estimation (see above), the cell free culture supernatants were subjected to protein precipitation with trichloroacetic acid. Precipitated proteins were dissolved in one tenth of the volume of Laemmli-buffer and 10 µl were analysed via SDS-PAGE. The StrepII-SUMO-PhoA protein is labelled with a star (*). **d** Western blot analysis of culture supernatants (CS) precipitated with TCA and whole-cell lysate samples (CE). Blots were developed with anti-PhoA antibodies. CS: 10 µl of a 10-fold dilution of the samples used for Coomassie staining (see above) were loaded; CE: 10 µg whole-cell protein per lane

There are several reports that the SUMO-tag supplied with an N-terminal SEC signal peptide and C-terminally fused to different short polypeptides is efficiently secreted from *B. subtilis* cells [17–20]. Therefore, we chose SUMO additionally fused to a StrepII-tag at its N-terminus to promote secretion of POIs (Fig. 1). The StrepII-SUMO part was fused to the AmyQ signal peptide that originates from a *B. amyloliquefaciens* amylase and is encoded on plasmid pHT43. As a reporter to screen for expression and secretion efficiency, we used the *E. coli* alkaline phosphatase PhoA. PhoA is exclusively active when secreted, because correct folding is dependent on disulphide-bridge formation that does not occur in the reductive cytoplasmic environment [21]. Furthermore, PhoA activity can easily be screened in plate assays with BCIP (5-Bromo-4-chloro-3-indolyl phosphate) by means of blue colour intensity of single colonies (Fig. 2a). Colonies of the resulting *B. subtilis* strain KO7A/pJHS03 (compare Fig. 1a) grown on LB-plates with BCIP and IPTG displayed a faint light blue phenotype, most probably because the activity on plates remained at the detection limit. The culture supernatants of KO7A/pJHS03 exhibited clear PhoA-activity that was about 50-fold higher than the basal activity of the control strain KO7A/pJHS02 without cloned *phoA* (Fig. 2b). The AmyQ signal peptide-encoding region (SP*amyQ*) in pJHS03 was replaced by a library of 173 different *B. subtilis* signal peptide-encoding DNA fragments, and, for an additional control, with the AprE signal peptide-encoding region (SP*aprE*; pJHS04). The resulting plasmid library was transformed to *B. subtilis* KO7A and plated on BCIP and IPTG containing medium. Several single colonies with a distinct blue phenotype were obtained (Fig. 2a), the plasmids of which were re-isolated and subjected to DNA-sequencing. In parallel, the plasmids were retransformed to *B. subtilis* KO7A and checked for restoration of the blue colony phenotype. In summary, three different clones were isolated, encoding the signal peptide of YopL (pJHS05), YncM (pJHS06) and YoaW (pJHS07). For PhoA activity in culture supernatants, the SP*yoaW* encoding strain KO7A/pJHS07 showed the best result of an approximately fivefold activity in relation to the SP*amyQ* encoding strain KO7A/pJHS03 (Fig. 2b). The SP*aprE* encoding control strain KO7A/pJHS04 displayed only half the PhoA activity of SP*amyQ encoding* KO7A/pJHS03. A distinct band in the range of the calculated molecular weight of 59 kDa of the StrepII-SUMO-PhoA fusion protein could be detected in SDS-PAGE of culture supernatants (Fig. 2c). The amount of that protein correlated with the measured PhoA-activity. In Western Blots developed with anti-PhoA antibodies it became clear that this protein represents the StrepII-SUMO-PhoA-6xHis fusion protein (Fig. 2d), most of which is located in the culture supernatant as a soluble protein and smaller amounts remaining cell associated. Taken together, the expression and/or secretion efficiency of the fusion reporter is clearly dependent on the individual signal peptide, and SP*yoaW* resulted in highest yields.

### Evaluation of the newly constructed secretion expression system

**Fig. 3:**
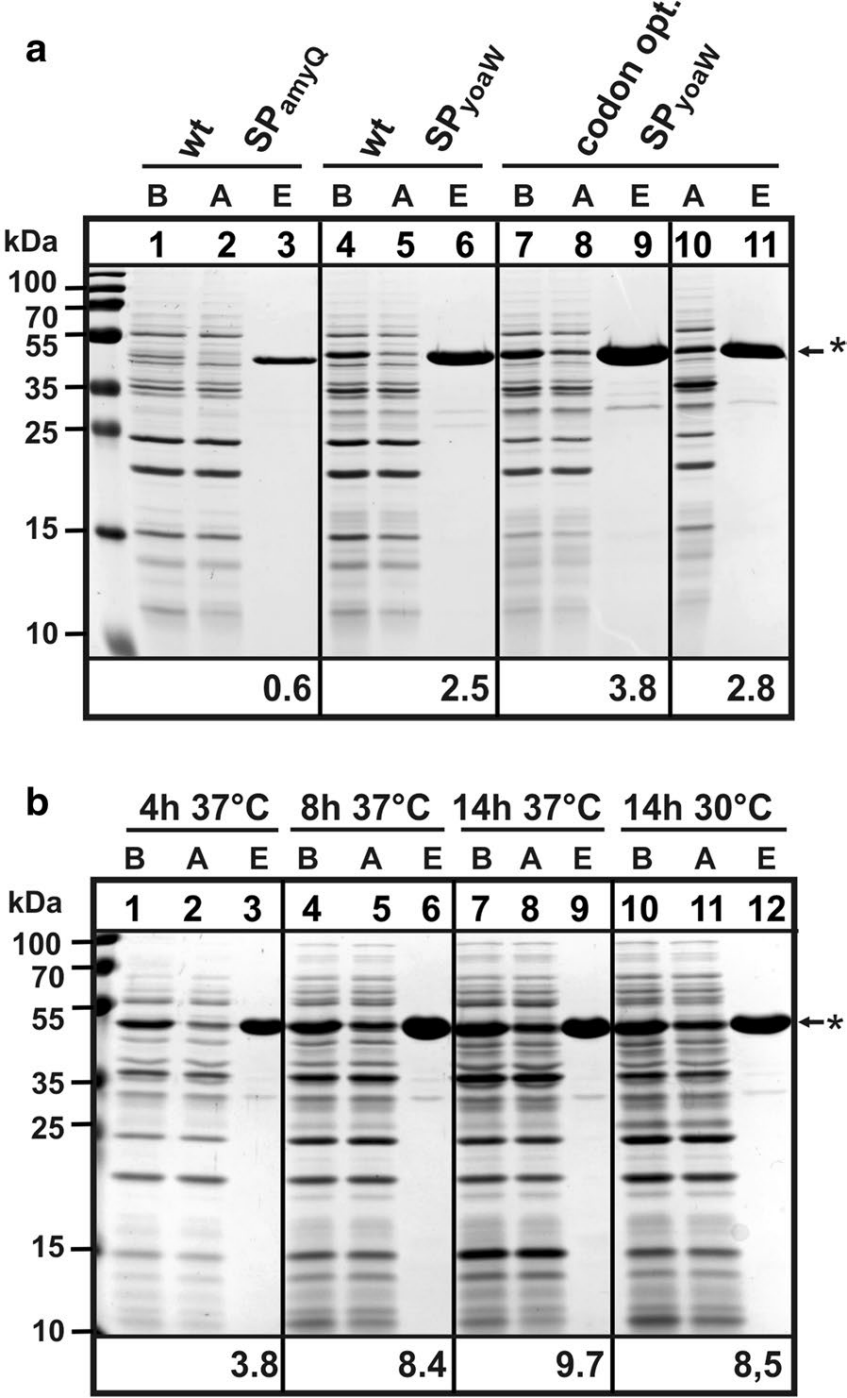
SDS-PAGE analysis of scFv production in *B. subtilis* using the SUMO fusion system. **a** Comparison of secretory production of the StrepII-SUMO-scFv fusion protein with the AmyQ signal peptide (SP*amyQ*) and the YoaW signal peptide (SP*yoaW*). Liquid cultures in LB-medium were incubated in a 37 °C water bath under rigorous shaking. IPTG was added to a final concentration of 1 mM at an OD_600_ of 0.8, and cultures were incubated for further 4 h. Lanes 1–3: *B. subtilis* KO7A/pJHS08 (SP*amyQ*), lanes 4–6: KO7A/pJHS10 (SP*yoaW*), lanes 7–11: KO7A/pJHS11 (SP*yoaW*; SUMO and GFP-specific scFv encoding sequence codon optimized for *B. subtilis*). Lanes 1–9: cell free culture supernatant before (B) and after (A) incubation with NiNTA-agarose, and elution fraction (E) from NiNTA agarose. Lanes 10 and 11: NiNTA-agarose directly added to culture without removing cells by centrifugation. The StrepII-SUMO-scFv fusion protein is labelled with a star (*), and the amount of that protein purified via IMAC from 1 l of liquid culture is indicated below the lines of elution fractions (mg protein as estimated by a Bradford assay). **b** Analysis of secretory production of the StrepII-SUMO-scFv fusion protein with strain KO7A/pJHS11 (SP*yoaW*; SUMO and GFP-specific scFv encoding sequence codon optimized for *B. subtilis*) with cultivation at 30 °C and 37 °C, and IPTG induction for 4, 8, and 14 h. For further details see above

To evaluate versatility of a YoaW signal peptide-StrepII-SUMO fusion cassette for high secretory expression of POIs, a single-chain variable fragment antibody against the green fluorescent protein (GFP-specific scFv) protein was used first. The variable fragment encoding DNA-sequence derived from a cDNA library generated from mRNA isolated from chicken spleen that had been immunized with GFP. The cDNA library was cloned to a phagemid vector and GFP-specific scFv variants were isolated by a standard phage-display screen, one of which was chosen to construct a YoaW signal peptide-StrepII-SUMO-scFv-6xHis fusion (JHS10, Fig. 1a). A respective fusion with the AmyQ signal peptide served as a control (JHS08, Fig. 1a). A faint band at the calculated molecular weight of the StrepII-SUMO-scFv-6xHis fusion protein could be detected in SDS-PAGE analysis of culture supernatants of *B. subtilis* KO7A/pJHS08 encoding SP*amyQ* after induction with 1 mM IPTG for 4 h at 37 °C (Fig. 3a, lane 1). This protein bound to NiNTA-agarose (Fig. 3a, lane 2) and could then be affinity-purified under native conditions to yield 0.6 mg protein per liter of culture supernatant. In comparison, KO7A/pJHS010 encoding SP*yoaW* produced distinctly more of the extracellular fusion protein and resulted in 2.5 mg protein per liter of culture supernatant (Fig. 3a, lanes 4–6). These data show that for both, PhoA and GFP-specific scFv, secretory expression efficiency is improved by a factor of four to five by replacing SP*amyQ* with SP*yoaW*. It also shows that the secretory expression efficiency depends on the type of signal peptide N-terminally fused to the StrepII-SUMO-tag, but seems not to depend on the type of the carried protein C-terminally fused to the StrepII-SUMO-tag. To further improve expression of the fusion protein, the SUMO-scFv encoding region of plasmid pJHS10 was codon-optimized for *B. subtilis,* resulting in fusion construct JHS11 (Fig. 1a). When compared to *B. subtilis* KO7A/pJHS08, production of the fusion protein is elevated more than sevenfold with *B. subtilis* KO7A/pJHS11 (Fig. 3a, lines 7–9).

To confirm these results, a third example of a POI that represents a hydrolytic *Bacillus* secretory enzyme was cloned to the StrepII-SUMO vectors. The *B. amyloliquefaciens* extracytoplasmic RNase barnase was chosen, which had been analysed for secretion in *B. subtilis* earlier [17]. The pro-barnase encoding region was translationally fused to the SP*amyQ* and the SP*yoaW* encoding plasmids resulting in fusion construct JHS13 and JHS14, respectively (Fig. 1a). To avoid toxic effects of barnase expression, it was cloned together with its inhibitor barstar. The C-terminal His-tag fusion to barnase was omitted. Again, an at least fivefold increase in secretory expression could be obtained with the SP*yoaW* construct of strain KO7A/pJHS14 in comparison to the SP*amyQ* fusion of strain KO7A/pJHS14 (Additional file 2: Fig. S2A).

### Optimization of culture conditions and evaluation of purification strategy

**Fig. 4:**
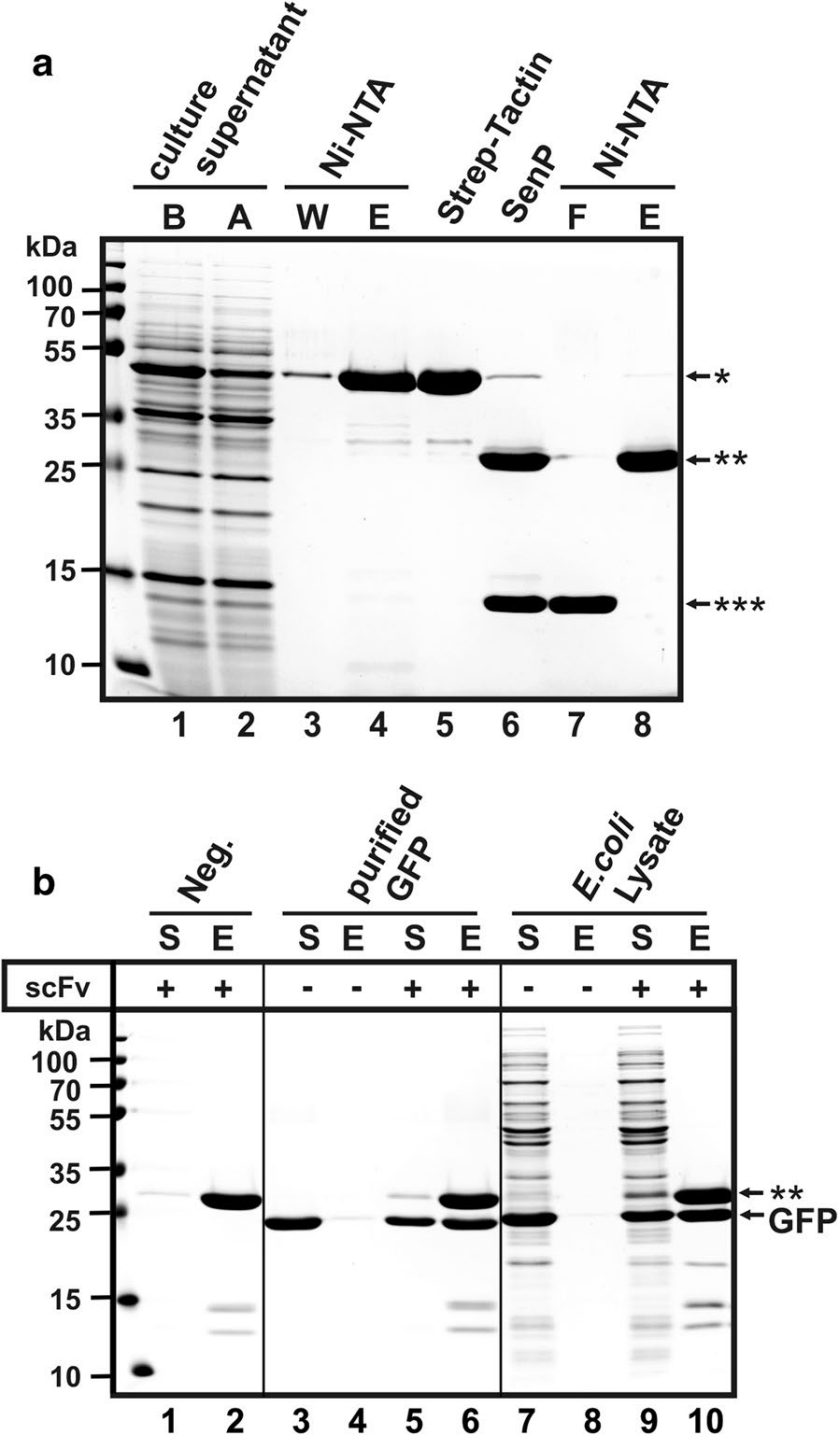
GFP-specific scFv purification and antibody-antigen binding assay. **a** SDS-Page representing purification steps for the GFP-specific scFv antibody fragment from culture supernatants of *B. subtilis* strain KO7A/pJHS11, including immobilized metal ion affinity (IMAC) purification via the polyhistidine-tag and NiNTA, affinity purification via the StrepII-tag and StrepTactin agarose, and removal of the StrepII-SUMO part via SenP protease treatment. Cells were induced with IPTG at an OD_600_ of 0.8 and incubated for further 14 h at 30 °C. StrepII-SUMO-scFv fusion protein is marked with one star (*), GFP-specific scFv is marked with two stars (**), and StrepII-SUMO is marked with three stars (***). Lane 1 and 2: culture supernatant concentrated by TCA precipitation before (B) and after (A) NiNTA-agarose treatment. Lane 3: sample of wash fraction from first IMAC (W); lane 4: sample of elution fraction from first IMAC (E); lane 5: sample of elution fraction from StrepII-tag affinity purification; lane 6: sample of elution fraction from StrepII-tag affinity purification incubated with SenP-protease for 1 h at 37 °C; lane 7: sample of flow-through fraction of second IMAC (F); lane 8: sample of elution fraction from second IMAC (E). For details, see “Methods” section. **b** SDS-Page analysis of pull down assay using NiNTA magnetic agarose beads. GFP-specific scFv antibody fragments were bound to magnetic beads via the C-terminal polyhistidine tag (lanes are marked with a plus: +). Unloaded magnetic beads served as a control (marked with a minus: −). GFP was then added to the magnetic beads in excess as a purified native protein (lanes 3–6) or as a cleared lysate from *E. coli* cells overproducing GFP (lanes 7–10). As a negative control, no GFP was added (lanes 1 and 2). After 1 h incubation at 4 °C allowing interaction complex formation, the magnetic beads were removed, and a sample of each remaining supernatant (S) submitted to SDS-Page (lanes 1, 3, 5, 7 and 9). The beads were then washed and protein was eluted using 250 mM imidazole. The elution fractions (E) were submitted to SDS-Page (lanes 2, 4, 6, 8 and 10). GFP-specific scFv is marked with two stars (**)

The fast and easy production of highly pure proteins with the secretory expression system described above was exemplified with the StrepII-SUMO-scFv-6xHis fusion protein for standard laboratory equipment (water bath shaker and Erlenmeyer flasks) and standard LB-medium. A maximum yield for the GFP-specific scFv fusion protein was recovered when cells of *B. subtilis* KO7A/pJHS11 were induced at an OD_600_ of 0.8 with 1 mM IPTG (final concentration) and were then further incubated at 37 °C under rigorous shaking for 14 h (Fig. 3b). After the first affinity purification step via IMAC on NiNTA-agarose beads, 9.7 mg of fusion-protein per liter of culture supernatant was obtained. The StrepII-SUMO-scFv-6xHis fusion protein was contaminated by only small amounts of proteins at a molecular weight of 30 kDa, 10 kDa and below (Fig. 4a, lane 4). The StrepII-SUMO-scFv-6xHis fusion protein can also directly be trapped and purified quickly from cultures via IMAC without removing cells, simply by adding NiNTA agarose to the induced cultures 1 h before harvesting, and then separating cells from agarose beads (45–165 µm beadsize) by vacuum-filtration, either through standard cellulose-filter paper (e.g. Whatman grade 40; 8 µm particle retention) or through a frit of appropriate pore size (e.g. in Qiagen polypropylene columns for gravity-flow chromatography with Ni–NTA agarose). However, omitting the centrifugation step to get a cell-free supernatant resulted in a 25% reduction of the amount of fusion protein after the first IMAC step (Fig. 3, lanes 7–11). After a second affinity purification step via the N-terminal StrepII-tag using streptactin-agarose, the small molecular weight contaminations at 10 kDa and below were removed (Fig. 4a lane 5). The fusion protein was then processed with SenP2-protease (Fig. 4a, lane 6) and scFv-6xHis was separated by a second IMAC on NiNTA-agarose at a yield of 4.1 mg per 1 l of culture (Fig. 4a, lane 8). To show that the antibody fragment was purified as a native protein, binding to GFP was monitored in pull down assays with magnetic NiNTA agarose beads loaded with scFv-6xHis (Fig. 4b, lanes 1 and 2). GFP, either as a purified protein (Fig. 4b, lanes 3–6) or from lysates of a GFP producing *E coli* strain (Fig. 4b, lanes 7–10), was efficiently bound to the scFv-6xHis loaded beads, as both proteins could be eluted in an apparently stoichiometric ratio (Fig. 4b lanes 5/6 and 9/10). For the negative control with NiNTA agarose beads not pre-loaded with scFv-6xHis, no GFP was retained (Fig. 4b lanes 3/4 and 7/8).

As a second example, the pro-barnase fusion protein was purified using streptactin affinity chromatography at a yield of 10.6 mg/l of culture supernatant (Additional file 2: Fig. S2B, lane 3). After SenP processing (Additional file 2: Fig. S2B, lane 4), pure pro-barnase was obtained at a concentration of 5.3 mg/l of culture supernatant (Additional file 2: Fig. S2B, lane 5). The purified enzyme was highly active in degrading RNA isolated from *B. subtilis* (data not shown).

## Discussion

*Bacillus subtilis* produces a variety of extracellular proteases, and one of the main constraints in using it as a host for secretory expression is the fast degradation of recombinant proteins. Therefore, strains with multiple deletions in genes encoding extracellular proteases were developed that significantly improve yields [2, 11, 13]. The first extremely useful and most common strain is the eightfold protease deletion strain *B. subtilis* WB800 [2] or its descendant WB800N [14]. WB800 contains a chloramphenicol resistance marker (*cat*) of unknown location, which was inactivated by an insertion-deletion mutation of a neomycin resistance gene (*neo*) to obtain WB800N. WB800N bears the risk that expression vectors that encode a chloramphenicol selection marker are able to integrate in its genome via homologous recombination. This can finally result in the loss of free plasmid and reduction of the expression level due to the absence of a gene-dosage effect. Moreover, the *B. subtilis* DNA-uptake system transports linear single stranded DNA [22] and plasmid propagation is dependent on the uptake of rare concatemeric forms that are able to recyclize via recombination [23]. Therefore, a replicative plasmid that encodes larger homologous regions to the *B. subtilis* genomic DNA might recombine with the chromosome and transfers a resistant marker without being propagated. As we sought to construct a stable and universal secretory expression system for *B. subtilis*, our initial aim was to identify the *cat::neo* site in the WB800N genome and to realize a clean deletion. DNA-sequencing revealed that the original *cat* marker of WB800 is placed in a pBR322-derived plasmid integrated in the chromosome to delete the *ispA* gene that encodes a major cytoplasmic protease of *B. subtilis*. The *ispA::cat* deletion [24] had originally been combined with an *nprE/aprE* deletion (strain KN2; [25]), which was then used in the course of constructing WB800 [26] without knowledge of a whole plasmid integrated (Sui Lam Wong, pers. comm.). Therefore, WB800 or WB800N might be problematic in propagation of shuttle-vectors, and the use of alternative strains meanwhile available for scientific purpose (D. Zeigler, unpublished: *Bacillus* Genetic Stock Center, strain KO7, BGSCID 1A1133; [13]: strain BRB08) is advisable.

The secretion efficiency of heterologous proteins expressed in *B. subtilis* significantly depends on the nature of the signal peptide [27]. An optimal balance between biosynthesis and the flow of the target protein through all stages of the *B. subtilis* secretion pathway have been proposed to be of crucial importance with respect to yield and quality of secreted heterologous proteins [28]. Therefore, high throughput methods have been developed to screen for an optimal signal peptide for the specific protein of interest [28–31]. However, the success to find an optimal signal peptide depends on extensive screening that is time-consuming, mostly requires specific and expensive lab equipment, and still relies on fortunate coincidence. The individual roles of mature protein and signal peptide in the efficiency of expression and protein secretion of heterologous proteins are not completely understood [32]. The signal peptide, e.g. does not only mediate interaction with the secretion apparatus, but also effects mRNA structure, translation and cytoplasmic folding kinetics [5]. It has been shown that the signal peptide and approximately the first 30 amino acid residues of the mature protein build an ‘export initiation domain’, both cooperatively interacting with the SEC-translocase in a specific manner to initiate secretion [33]. After being thread through the SEC translocation pore and processing of the signal peptide by signal peptidase, the folding process of the mature protein supports secretion [34]. Therefore, we envisaged an expression system where any POI can be fused to an N-terminal carrier protein which itself is secreted by an optimal signal peptide. After secretion, it should be possible to purify the fusion protein in a fast and easy way, and to remove the carrier protein by proteolytic processing. The SUMO protein was an ideal candidate for that purpose, as it is an established fusion-tag that enhances expression, folding and stability of proteins [15, 16], especially for heterologous proteins of pharmaceutical relevance like antibody fragments [35]. Furthermore, it has already been used for secretory expression in *B. subtilis,* especially for small peptide antibiotics [17–20]. The SUMO tag can be processed proteolytically by the corresponding SenP-protease, which recognizes the three dimensional structure of SUMO and merely requires two glycine residues at the processing site [36]. For fast purification, we added a StrepII-affinity tag to the N-terminus of SUMO and included the possibility to connect the POI to a C-terminal 6xHis-tag. We refrained in using the 6xHis-tag directly following the signal peptide, as it can drastically reduce secretion efficiency [33].

By screening a library of 173 *B. subtilis* signal peptides, we found the YoaW signal peptide as most efficient in secretion of a StrepII-SUMO-PhoA-6xHis fusion protein. SP*yoaW* had been found earlier to increase secretory expression of *B. amyloliquefaciens* subtilisin by a factor of four when compared to the wild-type signal peptide encoding region [30]. However, SP*yoaW* did not represent the optimal sequence for subtilisin. Also, SP*yoaW* appeared as one of the worst signal peptide encoding DNAs for secretory expression of a *B. subtilis* aminopeptidase [37] and did not come up in screens with other target proteins, corroborating that it specifically improves secretory expression of the StrepII-SUMO-PhoA fusion protein. Because SP*yoaW* did also enhance secretory expression of a GFP specific scFv and of *B. amyloliquefaciens* barnase by a factor of more than four in comparison to SP*amyQ*, it most probably optimises secretory expression of the StrepII-SUMO-tag, irrespectively which protein is connected to the C-terminus of it. Interestingly, the second best signal peptide encoding region identified in our screen was SP*yncM*, which has been found as the best candidate in a screen with an aminopeptidase as a reporter [37].

The final yield of about 10 mg of fusion proteins and 4 mg highly pure protein after SenP-processing of the StrepII-SUMO part per liter of culture supernatant is in an upper range for secretory expression of heterologous proteins in *B. subtilis*. However, values of 10 to 15 mg/l of a fibrin specific single chain antibody [2] and of 130 mg/l of a D1.3 lysozyme specific His-tagged scFv have been reported [38]. Nevertheless, direct comparison of concentrations and yields generated with different methods are difficult, and a comparison of patterns and band intensities of secreted proteins in SDS-PAGE analyses might relativize results reported. The optimisation of growth medium and fermentation strategies will additionally be able to enhance yields significantly, e.g. by high cell-density fermentation [39]. Also, co-expression of chaperones and overexpression or deletion of other secretion specific factors might improve expression rates [40–42].

## Conclusions

In summary, plasmid vector pJHS12 and the purification strategy for secretory production of heterologous proteins with *B. subtilis* employing the YoaW signal peptide-StrepII-SUMO fusion might become an important and versatile system for the *B. subtilis* toolbox for recombinant protein recovery. It may also help to develop a generalized fed-batch operation strategy for high level secretory production of many proteins of pharmaceutical interest [43].

## Methods

### Standard molecular and microbiological techniques

Bacterial strains as well as plasmids used in this study are listed in Table 1. *E.* c*oli* and *B. subtilis* strains were grown aerobically at 30 °C or 37 °C in Luria–Bertani medium (LB). When necessary, LB was supplemented with ampicillin (100 µg/ml), chloramphenicol (10 µg/ml), kanamycin (10 µg/ml) or hygromycin (50 µg/ml). DNA manipulation and cloning-techniques were performed according to standard procedures [44] using *E. coli* NEB®-Stable (New England Biolabs Inc.). For standard plasmid DNA preparations, the PureYield™ Plasmid Miniprep or Midiprep Kits (Promega) were used. Alternatively, cells were suspended in CRA-buffer containing 1 mg/ml of lysozyme and incubated for 30 min at 37 °C when plasmid-DNA was isolated from *B. subtilis* cells. DNA sequencing was carried out by LGC-Genomics GmbH (Berlin, Germany) with appropriate sequencing primers. Sodium dodecyl sulfate–polyacrylamide gel electrophoresis (SDS-PAGE) was performed according to the method of Laemmli [45] and gels were stained with Coomassie Brilliant Blue. To analyze culture supernatants via SDS-PAGE, proteins were precipitated with trichloroacetic acid at a final concentration of 10% by incubation on ice for 1 h. After centrifugation at 12.000 rpm for 15 min at 4 °C, the protein sediment was washed with 1 ml of ice-cold acetone and again centrifuged for 15 min. The pellet was then air-dried, suspended in 200 µl of 1× Laemmli buffer and heated to 95 °C for 5 min. Western blotting was performed as described previously [46] using a semi-dry blotting procedure (Biorad, Trans-Blot Turbo). Blots were developed with polyclonal antibodies against *E. coli* alkaline phosphatase PhoA (OriGene Technologies, Inc.) at a dilution of 1:5000 and polyclonal secondary anti Rabbit-HRP antibodies (Carl Roth, #4750.1) according to the manufacturer’s specifications. Bands were visualized using the Clarity Western ECL chemiluminescence reagent (BioRad) and a gel documentation system (ChemiDocMP, Biorad).

**Table 1:** Strains and plasmids used in this study

Strain or plasmid	Relevant characteristics	Source or reference
*E. coli*		
Stable	F’ proA + B + lacI^q^ ∆(lacZ)M15 zzf::Tn10 (Tet^R^) ∆(ara-leu) 7697 araD139 fhuA ∆lacX74 galK16 galE15 e14-Φ80dlacZ∆M15 recA1 relA1 endA1 nupG rpsL (Str^R^) rph spoT1 ∆(mrr-hsdRMS-mcrBC)	New England Biolabs
C43(DE3)	F^–^ *ompT gal dcm hsdS*_*B*_(r_B_^−^ m_B_^−^) (DE3)	[55]
*B. subtilis*		
WB800N	nprE aprE epr bpr mpr::ble nprB::bsr∆vpr, wprA::hyg pB-cat5-neo-cat3 (Neo^R^)	[14]
KO7	∆*nprE,,*∆*aprE,* ∆*epr,* ∆*mpr,* ∆*nprB,* ∆*vpr,* ∆*bpr*	Zeigler, D. R.; Bacillus Genetic Stock Center, Columbus, OH, USA
KO7A	KO7, ∆*wprA* (Hyg^R^)	This work
Plasmids		
pBE-S	Vector used with the *B. subtilis* Secretory Protein Expression System, Km^R^, Amp^R^	TaKaRa/Clontech
pET11M-*senP2*	Expression plasmid for SenP2 protease	H. Besir, EMBL Protein Expression and Purification Core Facility, Heidelberg, Germany
pETM11-SUMO3GFP	T7 Expression vector for *E. coli* encoding His6-SUMO3-eGFP fusion protein, Kan^R^	H. Besir, EMBL Protein Expression and Purification Core Facility, Heidelberg, Germany
pET52B-*senP2*	SenP2 encoding region N-terminally fused to StrepII-tag	This work
pHT01	IPTG-inducible expression vector for *B. subtilis* carrying Pgrac and Cm^R^	[49]
pJH-scFv	Phagemid cassette vector, IPTG-inducible expression of anti-GFP ScFv-pIII fusion protein, Amp^R^	This work
pJHS01	pHT01 cured for MluI sites	This work
pJHS02	pJHS01 encoding fusion protein consisting of AmyQ signal peptide, StrepII-tag, SUMO-tag and 6xHis-tag	This work
pJHS03	pJHS02 with cloned *phoA*	This work
pJHS04-07	pJHS03 with signal peptide encoding region changed (SP*aprE* (04), SP*yncM* (05), SP*yopL*,(06) and SP*yoaW* (07))	This work
pJHS08	pJHS02 with cloned GFP-specific scFv gene	This work
pJHS09	pJHS02 with SP*amyQ* changed for SP*yoaW*	This work
pJHS10	pJHS09 with cloned GFP-specific scFv gene	This work
pJHS11	pJHS09 with codon usage optimized SUMO-scFv fragment	This work
pJHS12	pJHS09 with codon-usage optimized SUMO-cassette	This work
pJHS13	pJHS02 with cloned barnase and barstar	This work
pJHS14	pJHS12 with cloned barnase and barstar	This work
pPA4	vector with cloned *phoA*	[50]
pQEGFP	pQE30 (Qiagen) with cloned *gfp*	[53]

*SP* signal peptide sequence

### Measurement of alkaline phosphatase activity

Alkaline phosphatase (PhoA) activity of single colonies on solid medium was monitored by spreading of 5-bromo-4-chloro-3-indolyl phosphate (BCIP; 50 µg/ml final concentration) prior to plating. To quantify extracellular alkaline phosphatase activity in liquid culture, *B. subtilis* cells were incubated in LB + chloramphenicol under rigorous shaking at 37 °C. At an optical density OD_600_ of 0.8, IPTG was added to a final concentration of 1 mM. 4 h after induction, cells were harvested and alkaline phosphatase activity of the culture supernatants was determined as described elsewhere [47]. To subtract background activity, samples of *B. subtilis* strain KO7A/pJHS02 served as blanks.

### Whole genome sequencing of *B. subtilis* WB800N and construction of *B. subtilis* KO7A

Genomic DNA of *B. subtilis* strain WB800N was isolated according to a standard procedure [48] and 1 µg was sonographically fragmented using a Covaris S2 system (Woburn, United States) to generate a 200 bp-fragment library with the Ion Plus fragment library kit (Thermo Fisher Scientific, Darmstadt). Emulsion PCR was performed using the Ion PGM Hi-Q View OT2 kit. The enriched Ion Sphere Particles were then sequenced using an Ion Torrent PGM system and the Ion PGM Hi-Q Sequencing 200 kit with an Ion 316v2 chip according to the manufacturer’s manual (Thermo Fisher Scientific, Darmstadt). Raw data were exported as fastq and analyzed using Geneious R10 (Biomatters Ltd, Auckland, New Zealand). Read sequences were mapped to the reference genome of *B. subtilis* 168 (NC_000964.3) using the Geneious mapper with low sensitivity. The genome of WB800N was also *de*-*novo* assembled using MIRA4 (Chevreux) integrated in Geneious R10 to evaluate potential additional contigs compared to the reference strain.

*Bacillus subtilis* KO7 (*Bacillus* Genetic Stock Center (BGSC) Accession 1A1133) had been generated by sequentially knocking out the coding sequences of seven extracellular proteases. The deletion strain is free of antibiotic resistance genes or integrated plasmids (DR Zeigler, unpublished). To additionally delete the *wprA* gene, the *wprA::hyg* cassette of *B. subtilis* WB800N, where *wprA* is inactivated by an indel mutation of a hygromycin resistance marker (*hyg*), was transferred to KO7 via homologous recombination. A *wprA::hyg* DNA fragment was PCR-amplified with primer 1 and 2 (for all oligonucleotide primers refer to Additional file 3: Table S1) and genomic DNA of WB800N. The PCR product was transformed to *B. subtilis* KO7 with selection on LB + hygromycin. Correct integration of the *hyg* marker was checked by PCR, resulting in strain KO7A.

### Construction of plasmid backbone and PhoA-reporter fusion

All plasmid constructs are schematically drawn in Fig. 1a. Plasmid pJHS03 encoding a protein consisting of the *B. amyloliquefaciens* amylase (AmyQ) signal peptide fused to the StrepII-tagged SUMO protein and the C-terminally His6-tagged PhoA, was constructed by using the framework of plasmid pHT01 [49]. For compatibility with the TaKaRa/Clontech *B. subtilis* Secretory Protein Expression System (Cat. #3380) that enables the exchange of the signal peptide with a library of 173 *B. subtilis* signal peptides via recombineering, we first had to cure pHT01 for its two MluI restriction sites. To that purpose, the 1.2 kb fragment flanked by MluI was PCR amplified with primers 3 and 4 that replace MluI with PauI, which generates identical cohesive ends. The PauI restricted PCR product was then ligated to the MluI restricted vector fragment of pHT01. The absence of MluI in the resulting plasmid pJHS01 was proven by restriction analysis and DNA-sequencing with primer 5. An in vitro synthesized gene-fragment (Invitrogen GeneArt Gene Synthesis, Thermo Fischer) that encodes promotor, ribosome binding site, AmyQ signal peptide, StrepII-tag, SUMO and a 6xHis-tag (Additional file 4: Fig. S3A) was then inserted in the KpnI/SmaI restriction sites of pJHS01, yielding plasmid pJHS02 (Fig. 1b). For the SUMO-tag, the sequence of human SUMO3 of plasmid pETM11-SUMOGFP (EMBL Protein Expression and Purification Core Facility, Heidelberg, Germany) was used. The 0.8 kb KpnI/SmaI insert in pJHS02 was verified by DNA-sequencing with forward primer 6 and reverse primer 5.

Finally, a *phoA* encoding fragment, which was PCR amplified using primers 7 and 8 and plasmid pPA4 [50] as a template, was cloned to pJHS02 using the BamHI and SmaI restriction sites, resulting in plasmid pJHS03, which was again checked by DNA-sequencing with primers 5 and 6.

### Screening of a signal peptide library

The exchange of the AmyQ signal peptide in pJHS03 with a library of 173 *B. subtilis* signal peptides was performed using the TaKaRa/Clontech ‘*B. subtilis* Secretory Protein Expression System’ and the TaKaRa/Clontech ‘In-Fusion^®^ HD Cloning Kit’ according to the manufacturer’s instructions. In modification, we used reporter plasmid pJHS03 instead of the kit-included vector pBE-S. In brief, a plasmid library was generated by using the Eco52I and MluI restricted and purified pJHS03 vector fragment together with the mixture of signal peptide encoding DNA (SP-DNA) in the recombineering reaction. The subtilisin E signal peptide encoding region (SP*aprE*) served as a positive control. To that purpose, a PCR fragment generated with primers 9 and 10 and pBE-S as a template was used instead of the SP-DNA. The reaction mixtures were transformed to competent *E. coli* NEB®-Stable with plating on LB + ampicillin. Cells of more than 2000 single colonies were washed off the transformation plates and used for plasmid preparation. The plasmid library (SP-DNA) and the positive control (SP*aprE*) were then transformed to *B. subtilis* KO7A. Cells were plated on LB + chloramphenicol containing 1 mM IPTG and 50 µg/ml BCIP and incubated at 37 °C over night. Seven single colonies of the SP-DNA screen with a distinct blue colour from around 2500 colonies in total and two colonies of the SP*aprE* control were selected for further analysis. These individual clones were submitted to plasmid preparation, and respective signal peptide encoding regions were analyzed by DNA-sequencing. In summary, four different plasmids were obtained: pJHS04, pJHS05, pJHS06, and pJHS07 plasmids encoding signal peptides of AprE, YopL, YncM, and YoaW, respectively.

### Construction of GFP antibody fragment encoding plasmids

A gene encoding an scFv antibody against the green fluorescent protein (GFP-specific scFv) was isolated in a phage-display screen essentially as described earlier [51], using cDNA from spleen that originated from chicken immunized with GFP, and the surface expression phagemid vector pSEX81 (Progen Biotechnik GmbH, Heidelberg, Germany). The resulting plasmid pJH-scFv served as a template to amplify the GFP-specific scFv encoding DNA with primers 11 and 12. The PCR product was restricted with BglII and EcoRV and ligated to the BamHI and Eco47III digested vector fragment of pJHS02, yielding pJHS08. The SP*amyQ* region in pJHS02 was then exchanged with SP*yoaW* using the ‘In-Fusion^®^ HD Cloning Kit’ and pJHS07 and primers 9 and 10 as described above, resulting in pJHS09. Finally, GFP-specific scFv encoding DNA was cloned to the pJHS09 plasmid to obtain pJHS10 as described above.

To further improve expression, the SUMO-scFv DNA sequence was optimized for *B. subtilis* codon usage, which was accomplished by an in vitro synthesized gene-fragment (Invitrogen GeneArt Gene Synthesis, Thermo Fischer; Additional file 4: Fig. S3B). The DNA fragment was restricted with Eco52I and EcoRV and ligated to the pJHS09 vector fragment cut with Eco52I/Eco47III. Correct insertion of the DNA-fragment was verified by DNA-sequencing and the plasmid was named pJHS11.

To obtain a general cloning vector, the codon usage optimized StrepII-tag-SUMO encoding sequence of pJHS11 was PCR amplified with primers 13 and 14. The PCR product was ligated to the pJHS11 vector fragment, both restricted with Eco52I and Eco47III, resulting in plasmid pJHS12.

### Construction of barnase encoding plasmids

The *Bacillus amyloliquefaciens* ribonuclease barnase encoding gene (BAMF_RS37420) was cloned to plasmid pJHS02 and pJHS12 together with the barnase inhibitor barstar gene (BAMF_RS24500) in a two-step procedure. First, BAMF_RS24500 (barstar) was PCR amplified with primers 17 and 18 with genomic DNA of *B. amyloliquefaciens* DSM7 as a template. The resulting PCR product was restricted with BamHI/EcoRV and ligated to either pJSH02 or pJSH09 restricted with BamHI/Eco47III. The obtained plasmids were then used to clone the pro-barnase encoding region, which was amplified with primers 19 and 20 and genomic DNA of *B. amyloliquefaciens* DSM7 as a template. To that purpose, the BamHI and PstI restriction sites were used, resulting in plasmids pJHS13 and pJHS14 (Fig. 1a).

### Expression and purification of StrepII-tagged SenP-protease

In order to remove the StrepII-SUMO-tag from purified fusion proteins, the appropriate SenP-protease encoding gene was PCR amplified with primers 15, 16 and plasmid pETM11*senP2* (EMBL Protein Expression and Purification Core Facility, Heidelberg, Germany). The PCR product was restricted with SmaI/SacI and ligated to plasmid pET52B (Novagen) followed by transformation to *E. coli* BL21(DE3)-pLysS. The resulting plasmid was named pET52B*senP2*. The StrepII-tagged SenP2 protein was then purified from BL21(DE3)-pLysS/pET52B*senP2* according to a standard procedure (IBA Lifesciences, Göttingen, Germany), dialyzed against storage buffer (5 mM Tris–HCl, 75 mM NaCl, 2 mM DTT, 0,1 mM EDTA; 20% Glycerin, pH 7.5) and diluted to 125 ng/ml with the same buffer.

### Purification and quantification of GFP-specific scFv

All expression studies were performed with the *B. subtilis* eightfold extracellular protease knockout strain KO7A, to which respective expression plasmids were transformed via standard procedures. Cultures were grown in LB with shaking, induced with 1 mM IPTG at an OD_600_ of 0.8 and grown for additional 4 h, 8 h or 14 h. The cells were harvested by centrifugation at 6000 rpm for 30 min at 4 °C and the supernatant was used for further analysis or affinity‏ chromatography purification using Ni–NTA resin according to the QIAexpressionist protocol (QIAGEN, Hilden, Germany). All purification experiments were done under native conditions. In variation to the manual, to 1 l of cell-free supernatant 4 ml 50% NiNTA equilibrated in lysis-buffer was added, and the suspension was stirred for 1 h at 4 °C. The resin was recovered by filtration on Whatman filter paper, resuspended in an equal volume of lysis-buffer and then loaded on a gravity-flow polypropylene column (Qiagen, Cat No. 34964). After washing with two column volumes (cv) of wash-buffer, elution was performed with 5 cv of elution buffer and 1 ml fractions were collected. Protein containing fractions were pooled and subjected to streptactin affinity purification using 3 ml of Strep-Tactin XT Superflow resin (IBA Lifesciences, Göttingen, Germany) according to the manufacturer’s protocol. Protein containing elution fractions were again pooled, 1/10 the volume of SenP-protease (125 ng/ml) was added, followed by incubation at 37 °C for 1 h. Finally, the reaction mixture was submitted to a second IMAC using a 1 ml HisTrap HP column (GE Healthcare, Cat No. 17524701) and an ÄKTA-purifier FPLC system according to standard protocols. The native GFP-specific scFv containing elution fractions were pooled and buffer was changed to PBS buffer (pH 7.4) by ultrafiltration using an Amicon Ultra-15 10 K (Merck Millipore, Cat No. UFC901024) centrifugal device. Protein concentration was measured by a standard procedure [52] and for the purified protein spectrophotometrically at 280 nm with a calculated molar extinction coefficient for GFP-specific scFv of 35.870 M^−1^ cm^−1^.

### Immunoaffinity binding assay

To analyze functionality of the *B. subtilis* expressed and purified GFP-specific scFv antibody fragment, pull down experiments were performed with native GFP protein and GFP-specific scFv immobilized on Ni–NTA magnetic agarose beads (Qiagen, Cat No. 36113) using a Dynabeads™ MPC™-S magnetic particle concentrator (Thermo-Fischer Scientific). Cell free extract of *E. coli* XL1Blue/pQE*gfp* that contains untagged GFP [53] was prepared according to a standard procedure (Qiagen, QIAexpressionist). Pure untagged GFP was isolated using *E. coli* C43(DE3)/pETM11-SUMO3GFP as described earlier [54, 55]. Ni–NTA magnetic agarose beads were equilibrated with binding buffer (50 mM NaH_2_PO_4_, 300 mM NaCl, 10 mM imidazole pH 8) to obtain 1 ml of a 5% suspension. 1 mg of purified GFP-specific scFv in PBS-buffer was mixed with 600 µl of the magnetic beads, washed three times with 1 ml of binding buffer and resuspended in 600 µl of binding buffer to yield a 5% suspension of scFv-loaded magnetic beads. To 200 µl of either loaded or unloaded beads, 1 ml purified GFP (0.5 mg/ml in binding buffer) or 1 ml GFP containing cell free extract (1 mg/ml in binding buffer) was added. As a control, the remaining 200 µl scFv-loaded magnetic beads were mixed with 1 ml of binding buffer. All reaction mixtures were incubated in 1.5 ml Eppendorf tubes at 4 °C for 1 h in an over-head shaker. The magnetic beads were separated and the supernatant fractions saved. Then, all samples were washed three times with 1 ml of washing buffer (50 mM NaH_2_PO_4_, 300 mM NaCl, 20 mM imidazole pH 8) and finally, treated with 1 ml elution buffer (50 mM NaH_2_PO_4_, 300 mM NaCl, 250 mM imidazole pH 8) for 5 min at room temperature in an overhead shaker. After separation using the magnetic beads, the elution fractions (‘E’) were analyzed together with the supernatant fractions (‘S’) via SDS-PAGE (Fig. 4b).

## Additional files

**Additional file 1: Fig. S1.** Secretory production and purification strategy for heterologous proteins employing a StrepII-SUMO fusion and an optimized signal peptide of the general secretory pathway (SEC) in *B. subtilis*.

**Additional file 2: Fig. S2.** SDS-PAGE analysis of secretory production of barnase in *B. subtilis* using the SUMO fusion system.

**Additional file 3: Table S1.** Oligonucleotides used in this study.

**Additional file 4: Fig. S3.** DNA-sequence of in vitro gene synthesis products.
