# Supplementary figures and images for: The YoaW signal peptide directs efficient secretion of different heterologous proteins fused to a StrepII-SUMO tag in Bacillus subtilis
Source: Microb Cell Fact. 2019 Feb 7;18:31. doi: 10.1186/s12934-019-1078-0 (PMC6366066; doi:10.1186/s12934-019-1078-0)

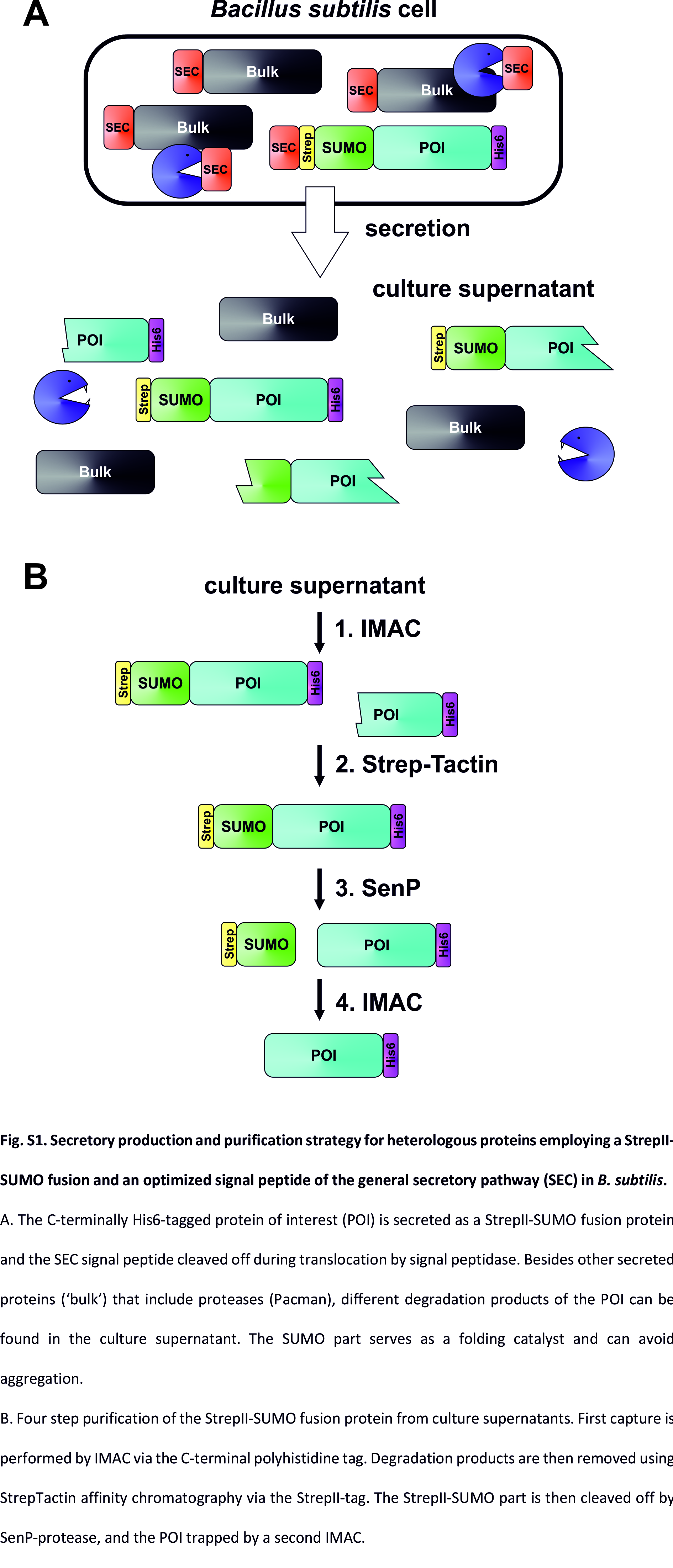

Supplement: Supplementary file 1 — Additional file 1: Fig. S1. Secretory production and purification strategy for heterologous proteins employing a StrepII-SUMO fusion and an optimized signal peptide of the general secretory pathway (SEC) in B. subtilis. [file 12934_2019_1078_MOESM1_ESM.jpg]

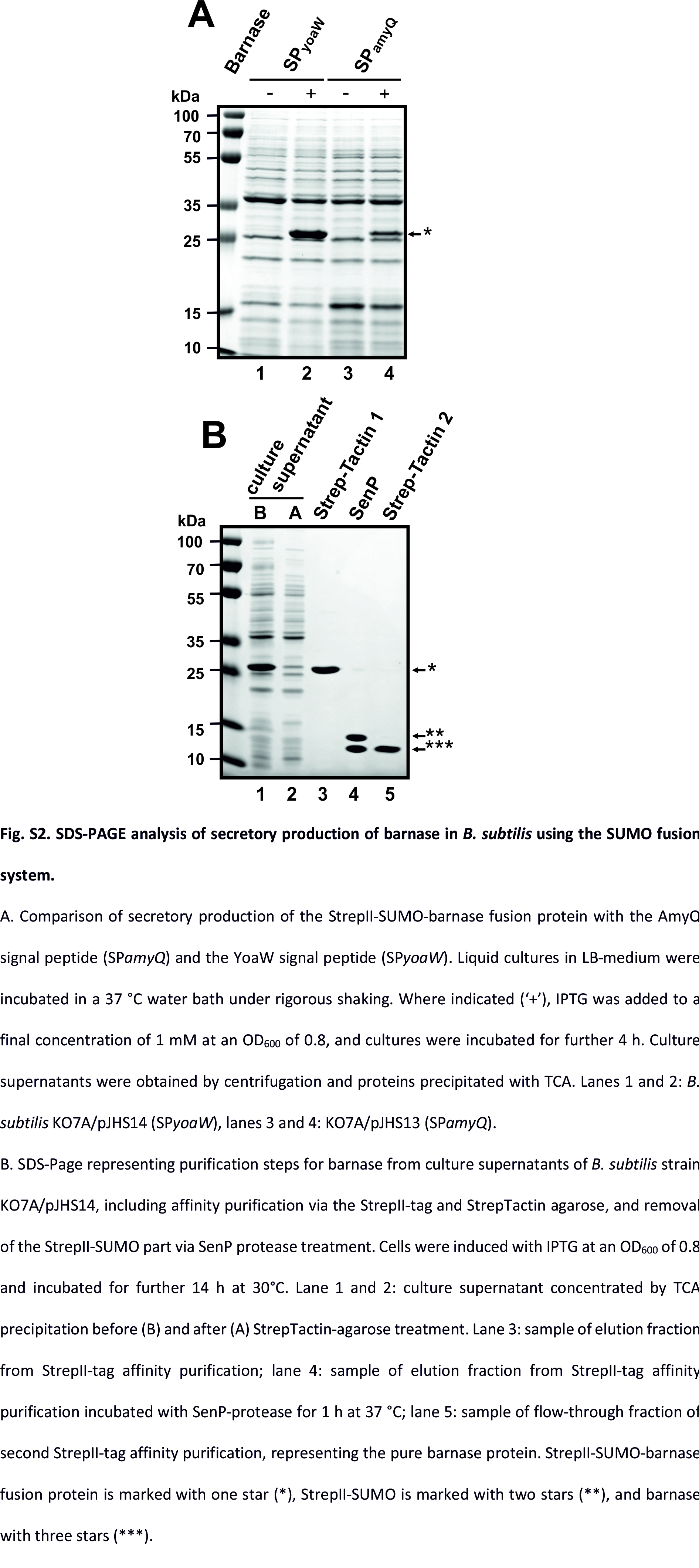

Supplement: Supplementary file 2 — Additional file 2: Fig. S2. SDS-PAGE analysis of secretory production of barnase in B. subtilis using the SUMO fusion system. [file 12934_2019_1078_MOESM2_ESM.jpg]

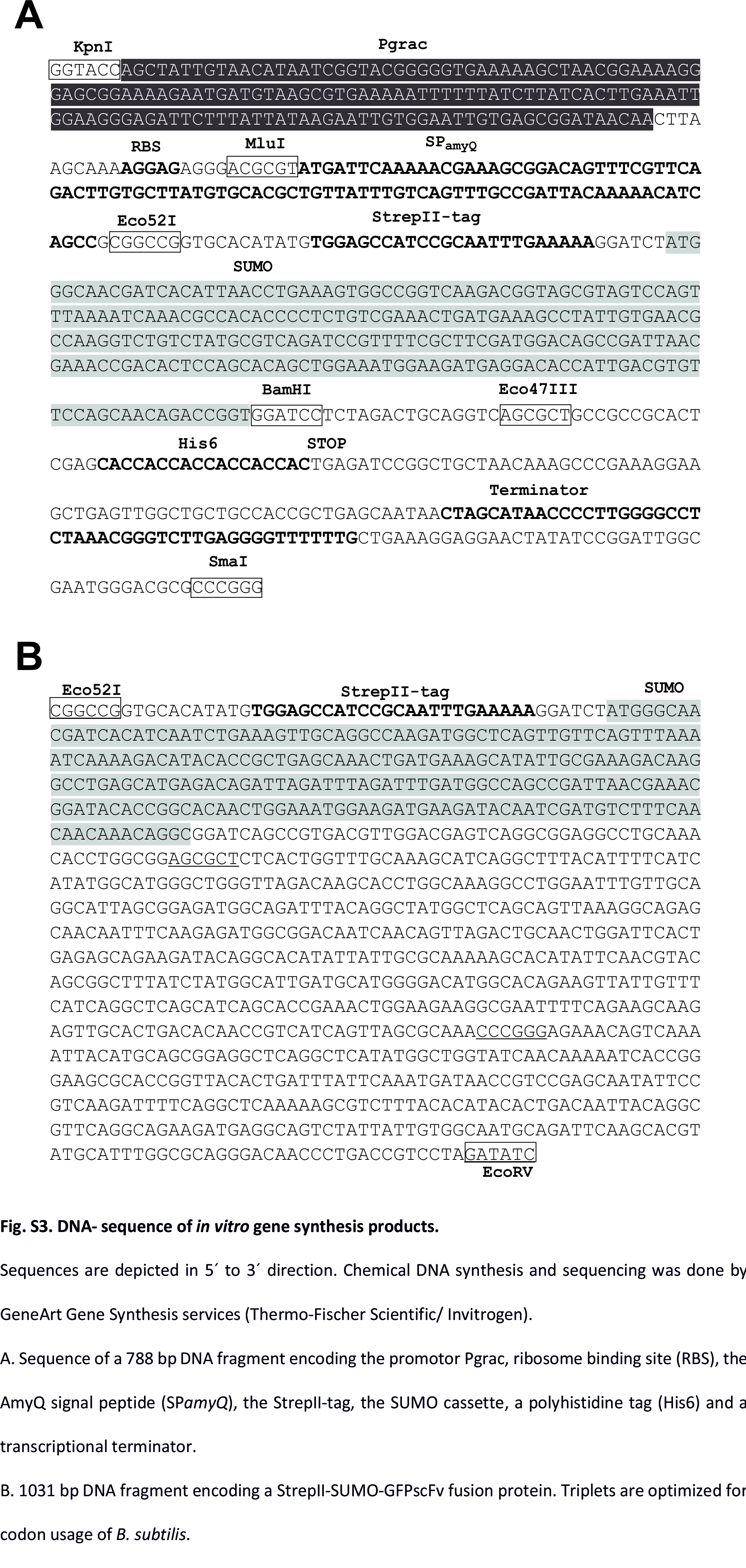

Supplement: Supplementary file 4 — Additional file 4: Fig. S3. DNA-sequence of in vitro gene synthesis products. [file 12934_2019_1078_MOESM4_ESM.jpg]
