# Supplementary material for: The YoaW signal peptide directs efficient secretion of different heterologous proteins fused to a StrepII-SUMO tag in Bacillus subtilis
Source: Microb Cell Fact. 2019 Feb 7;18:31. doi: 10.1186/s12934-019-1078-0 (PMC6366066; doi:10.1186/s12934-019-1078-0)
Supplement: Supplementary file 3 — Additional file 3: Table S1. Oligonucleotides used in this study. [file 12934_2019_1078_MOESM3_ESM.docx]

**Table S1.** Oligonucleotides used in this study.

| **No** | **Nucleotide sequence* in 5´-> 3´direction** | **Restriction site** | **remarks** |
| --- | --- | --- | --- |
| 1 | TGCGACACTATCCAAGATGTCCA |  | deletion of *wprA*, f |
| 2 | CCAGCTTGTTGACCTTAAGCTGTA |  | deletion of *wprA*, r |
| 3 | GGCCATGCGCGCTGCGCGAGAAGATTGTGCAC | PauI | MluI deletion, f |
| 4 | GGCCATGCGCGCGACGTGAAAAAAGCCCGCTCA | PauI | MluI deletion, r |
| 5 | TGTTTCAACCATTTGTTCCAGGT |  | pHT sequencing, r |
| 6 | AGCTATTGTAACATAATCGGTACG |  | pHT sequencing f |
| 7 | GGCCATGGATCCAACCGGGCTGCTCAGGGCGATA | BamHI | *phoA*, f |
| 8 | GGCCATCCCGGGTTTCAGCCCCAGAGCGGC | SmaI | *phoA*, r |
| 9 | CTTAAGCAAAAGGAGAGGGACGCGT | MluI | SP*aprE*, f (SP Kit) |
| 10 | ATGGCTCCACATATGTGCACCGGCCG | Eco52I | SP*aprE*, r (SP Kit) |
| 11 | GGCCATAGATCTGCCGTGACGTTGGACGAG | BglII | GFP-specific scFv, f |
| 12 | GGCCATGATATCTAGGACGGTCAGGGTTGTC | EcoRV | GFP-specific scFv, r |
| 13 | GGCCATCGGCCGGTGCACATATGTGGAGCCATCCGCAATTTGAAAAAG | Eco52I | StrepII-tag-SUMO, f |
| 14 | GGCCATAGCGCTGACCTGCTGCAGAGAGGATCCGCCTGTTTGTTGTTGAAAGACATCGATTGTA | Eco47III / PstI | StrepII-tag-SUMO, r |
| 15 | GGCCATCCCGGGATGGCAAGCGATCTGCTGGAA | SmaI | SenP, f |
| 16 | GGCCATGAGCTCGCCGCTACAGGGCGCGTCCCA | SacI | SenP, r |
| 17 | GGCCATGGATCCTAACTGCAGGGCAGAATCCGGCCATTTCTGA | BamHI / PstI | barstar, f |
| 18 | GGCCATGATATCTTAAGAAAGTATGATGGTGATGTCG | EcoRV | barstar, r |
| 19 | GGCCATGGATCCGGGATGCTGTTTTCAACAGCTGC | BamHI | barnase, f |
| 20 | GGCCATCTGCAGTTATCTGATTTTTGTAAAGGTCTGATA | PstI | barnase, r |

* restriction endonuclease recognition sites are underlined

Sequence orientation in 5´to 3´: f forward, r reverse
